# Systematic Review: microRNAs as Potential Biomarkers in Mild Cognitive Impairment Diagnosis

**DOI:** 10.3389/fnagi.2021.807764

**Published:** 2022-01-12

**Authors:** Natalia Ogonowski, Stefanny Salcidua, Tomas Leon, Nayaret Chamorro-Veloso, Cristian Valls, Constanza Avalos, Alejandro Bisquertt, Miguel E. Rentería, Paulina Orellana, Claudia Duran-Aniotz

**Affiliations:** ^1^Latin American Institute for Brain Health (BrainLat), Universidad Adolfo Ibanez, Santiago, Chile; ^2^Cognitive Neuroscience Center (CNC), National Scientific and Technical Research Council (CONICET), Universidad de San Andrés, Buenos Aires, Argentina; ^3^Faculty of Engineering and Sciences, Universidad Adolfo Ibanez, Santiago, Chile; ^4^Global Brain Health Institute, Trinity College, Dublin, Ireland; ^5^Memory and Neuropsychiatric Clinic (CMYN) Neurology Department, Hospital del Salvador, Faculty of Medicine, University of Chile, Santiago, Chile; ^6^Neurognos Spa, Santiago, Chile; ^7^Department of Genetics and Computational Biology, Queensland Institute of Medical Research (QIMR) Berghofer Medical Research Institute, Brisbane, QLD, Australia; ^8^Center for Social and Cognitive Neuroscience (CSCN), School of Psychology, Universidad Adolfo Ibanez, Santiago, Chile

**Keywords:** systematic review, microRNA, biomarkers, fluid biomarkers, diagnosis, mild cognitive impairment

## Abstract

The rate of progression from Mild Cognitive Impairment (MCI) to Alzheimer's disease (AD) is estimated at >10% per year, reaching up to 80–90% after 6 years. MCI is considered an indicator of early-stage AD. In this context, the diagnostic screening of MCI is crucial for detecting individuals at high risk of AD before they progress and manifest further severe symptoms. Typically, MCI has been determined using neuropsychological assessment tools such as the Montreal Cognitive Assessment (MoCA) or Mini-Mental Status Examination (MMSE). Unfortunately, other diagnostic methods are not available or are unable to identify MCI in its early stages. Therefore, identifying new biomarkers for MCI diagnosis and prognosis is a significant challenge. In this framework, miRNAs in serum, plasma, and other body fluids have emerged as a promising source of biomarkers for MCI and AD-related cognitive impairments. Interestingly, miRNAs can regulate several signaling pathways *via* multiple and diverse targets in response to pathophysiological stimuli. This systematic review aims to describe the current state of the art regarding AD-related target genes modulated by differentially expressed miRNAs in peripheral fluids samples in MCI subjects to identify potential miRNA biomarkers in the early stages of AD. We found 30 articles that described five miRNA expression profiles from peripheral fluid in MCI subjects, showing possible candidates for miRNA biomarkers that may be followed up as fluid biomarkers or therapeutic targets of early-stage AD. However, additional research is needed to validate these miRNAs and characterize the precise neuropathological mechanisms.

## Introduction

### Diagnostic Criteria in MCI

Diagnostic criteria for Mild Cognitive Impairment (MCI) have changed over time (Petersen, 2004; Winblad et al., 2004; Sachdev et al., 2015; Alzheimer's Association, 2018). At present, MCI is defined as a heterogeneous clinical syndrome which presents a significant change in cognitive function and deficits on neuropsychological testing but a relatively intact functionality (Winblad et al., 2004). The diagnosis of MCI depends on which cognitive and functional tests are used (Albert et al., 2011), these include the Mini-Mental Status Examination (MMSE; Arevalo-Rodriguez et al., 2021) or the Montreal Cognitive Assessment (MoCA; Nasreddine et al., 2005; Albert et al., 2011; Ciesielska et al., 2016). The presence or absence of MCI depends on the sensitivity and specificity of those tests and estimates of premorbid cognitive functioning. Appropriate and valid screening tests are crucial in evaluating patients with suspected MCI (Albert et al., 2011).

Currently, the MoCA is the recommended cognitive screening tool for MCI (Pinto et al., 2019). MoCA has a sensitivity between 80 and 100% and a specificity between 75 and 82% (Langa and Levine, 2014). However, the MoCA assessment varies depending on educational level, lifestyle factors, and ethnic diversities (Gagnon et al., 2013; O'Driscoll and Shaikh, 2017), limiting its use as a screening tool. Other cognitive assessments, including the MMSE and the Dementia Rating Scale (DRS), are not recommended as screening tools for MCI because of their specificity and sensitivity (Espino et al., 2001; Matallana et al., 2011). MoCA is currently more sensitive to diagnosing MCI than MMSE (Langa and Levine, 2014; Ciesielska et al., 2016).

The prevalence rate of MCI ranges from 6% (Sachdev et al., 2015) up to 25% for ages 80–84 (Livingston et al., 2017) in the population over 60 years of age. MCI is often considered a prodromal stage of dementia since neurodegenerative changes in the brain develop many years before symptoms are presented and dementia is diagnosed (Hulette et al., 1998). In long follow-up studies, annual rates of progression of MCI to dementia have been estimated at between 8 and 15% (Mitchell and Shiri-Feshki, 2009; Grill et al., 2013), which increases 80–90% after ~6 years (Petersen et al., 2001; DeCarli, 2003; Bruscoli and Lovestone, 2004; Petersen, 2004; Panza et al., 2005; Pinto and Subramanyam, 2009).

### Traditional and New Biomarkers in the Diagnosis of MCI

Since MCI is defined based on clinical criteria, biomarkers are not mentioned in its definition and diagnosis (Blennow and Hampel, 2003; Yin et al., 2013; Blennow and Zetterberg, 2018). However, based on recent research, biomarkers could offer support in identifying if the etiology of the MCI is AD-related or related to other pathologies, predicting the risk of progression to dementia, and helping in deciding the treatments options (Albert et al., 2011).

#### Biomarkers to Diagnose MCI Due to AD

Biomarkers for the diagnosis of MCI due to AD have not been validated yet. Most of them are based on the study of neuroimaging in AD (Yin et al., 2013) and the analysis of peripheral proteins in cerebrospinal fluid (CSF), including amyloid-beta (Aβ) and total (t-Tau) or phosphorylated (p-Tau) forms of Tau (Blennow and Zetterberg, 2018).

Neuroimaging techniques allow the evaluation of atrophy in medial temporal lobe regions, mainly the hippocampus and entorhinal regions, as well as the posterior cingulate cortex (Fennema-Notestine et al., 2009). In addition, neuroimaging could help to evaluate hypometabolism in the temporoparietal and posterior cingulate cortex (Kim et al., 2010; Habert et al., 2011). The techniques mainly used in neuroimaging are magnetic resonance imaging (MRI; Yin et al., 2013), fluorodeoxyglucose positron emission tomography (FDG-PET; Kim et al., 2010), and hypoperfusion of parietal cortices and the hippocampus as measured by single-photon emission computed tomography (SPECT; Habert et al., 2011). In addition, neuroimaging can assess Aβ accumulation using positron emission tomography using the (11)C-labeled Pittsburgh Compound-B ((11)C-PIB) ligand (Zhang et al., 2014).

On the other hand, lower concentrations of Aβ (Aβ_1−40_, Aβ_1−42_, and Aβ_1−42_/Aβ_1−40_ ratio) were detected in the CSF of patients with MCI, indicating higher concentrations of cerebral Aβ and progressive cognitive decline (Blennow and Hampel, 2003; Herukka et al., 2005; Okonkwo et al., 2010; Parnetti et al., 2012; Forlenza et al., 2015; Hansson et al., 2018). Similarly, the detection of t-Tau and p-Tau in CSF samples are also used to diagnose MCI and AD with 85% sensitivity and 80% specificity approximately, indicating neuronal damage and predicting the progression from MCI to AD (Trojanowski et al., 2010). The combination of Aβ_1−42_ and Tau biomarkers has demonstrated a high sensitivity of 95% and specificity of 83% (Hansson et al., 2006).

The use of these biomarkers is limited in the case of neuroimaging by the high cost and low availability and access to these technologies (el Kadmiri et al., 2018; Swarbrick et al., 2019). At the same time, the analysis of CSF biomarkers is limited by the difficulty of obtaining the samples, low patient acceptance rates, and high costs (Hampel et al., 2018). For these reasons, it is necessary to find easily accessible and inexpensive biomarkers, such as blood biomarkers (Hampel et al., 2018).

In this line, Aβ and Tau protein levels have been measured in the blood (plasma or serum) of MCI subjects (Thambisetty and Lovestone, 2010; Colijn and Grossberg, 2015; Janelidze et al., 2020). Studies of Aβ plasma have yielded contradictory results in MCI subjects. Some studies have shown increased Aβ_1−42_ levels in MCI and AD compared with controls (Giliberto et al., 2004; Chouraki et al., 2015; Hanon et al., 2018). In contrast, a decrease in plasma Aβ_1−42_ or Aβ_1−42_/Aβ_1−40_ ratio has been observed in the progression of healthy controls and MCI to AD (Graff-Radford et al., 2007; Seppälä et al., 2010; Fei et al., 2011; Chouraki et al., 2015). Similarly, plasma t-Tau some studies have also shown inconsistent results. Increased levels of t-Tau have been found in AD but not in MCI (Zetterberg et al., 2013; Mattsson-Carlgren et al., 2021). Additionally, in another study, t-Tau increases in MCI (Dage et al., 2016), or increases in both MCI and early AD (Chiu et al., 2014), or no changes in MCI or AD (Wang et al., 2016). Finally, levels of t-Tau have also been shown to decrease in MCI and AD (Larry Sparks et al., 2012). These discrepancies may be given by differences in the methodology for measuring biomarker levels, clinical factors referred to the characteristics of the subjects under study, and the lack of validation of brain accumulation of Aβ and Tau (Chen et al., 2019; Qu et al., 2021).

Plasma p-Tau has been validated with measurements of p-Tau in CSF and Tau-PET (Herukka et al., 2005; Dage et al., 2016; Barthélemy et al., 2020; Mattsson-Carlgren et al., 2021). Two phosphorylation epitopes, p-Tau181 and p-Tau217, have been extensively studied and described as biomarkers in MCI (Barthélemy et al., 2020; Mattsson-Carlgren et al., 2021). Two studies have shown that plasma p-Tau181 can predict AD and differentiate AD from other neurodegenerative diseases (Janelidze et al., 2020; Karikari et al., 2020). In a longitudinal study in MCI subjects, increased plasma levels of p-Tau217 have been correlated with cognitive impairment and increased brain atrophy (Mattsson et al., 2016; Janelidze et al., 2020), showing the same results which have also been observed in longitudinal studies of p-Tau in CSF (Falcon et al., 2018; Koychev et al., 2020). Additionally, another longitudinal study evaluating the same previously mentioned biomarkers in controls, MCI subjects, and AD patients demonstrated that plasma p-Tau, rather than plasma Aβ, is related to cognitive changes in the MCI stage (Chen et al., 2019).

The presence of biomarkers is a predictive factor for rapid progression to dementia, which includes (van Maurik et al., 2019): significant cerebral white matter hyperintensities (WMH), Apolipoprotein E4 (APOE4) carrier status, abnormal brain Aβ_1−42_ on positron emission tomography (PET) or CSF analysis, abnormal Tau on PET or CSF analysis (Dunne et al., 2021).

#### Biomarkers and Treatment

Currently, there is no specific pharmacological treatment for MCI (Kasper et al., 2020). However, recent studies have suggested that some MCI subgroups (defined by biomarkers) might benefit from the treatment of acetylcholinesterase inhibitors (AChEIs) or Memantine (Dunne et al., 2021). Notably, despite the lack of specific and FDA-approved treatment, over 60% of patients with MCI are prescribed of AChEIs after positive amyloid PET scans. On the other hand, 24% of MCI patients are recommended to receive counseling about safety and future planning (Makizako et al., 2016; Rabinovici et al., 2019).

Nevertheless, several pharmacological clinical trials have shown little to no cognitive improvement in patients with MCI, both for classical anti-dementia drugs (e.g., acetylcholinesterase inhibitors) and other medications (Russ and Morling, 2012; Kasper et al., 2020) thus the importance of non-pharmacological treatments has increased. Several trials are showing the impact of cognitive training (Hill et al., 2017), physical activity (Rovner et al., 2018) and multimodal interventions (Chandler et al., 2016) on cognitive and functional outcomes. Unfortunately, the impact of these treatments on dementia prevention is still unclear (Livingston et al., 2020; Dunne et al., 2021). In AD-related dementia, several non-pharmacological interventions have been evaluated for both cognitive (Loi et al., 2018) and non-cognitive symptoms (Abraha et al., 2017) however, due to methodological differences, there is still no clear evidence of its effectiveness (Wang et al., 2020).

Currently, several investigations aim to search for biomarkers in peripheral fluids that account for pathological changes in the brain during the development and course of AD (Irizarry, 2004), which also allow detection of the disease at earlier stages. Some peripheral biomarkers that have been studied included molecules such as proteins, peptides, nucleic acids, microRNAs (miRNAs), lipids, and metabolites, which can be detected in serum, plasma, cellular components, and exosomes (Thambisetty and Lovestone, 2010; Lista et al., 2013). Among them, levels of peripheral miRNAs have been found dysregulated in AD (Sheinerman et al., 2013; Roderburg and Luedde, 2014; Schwarzenbach et al., 2014; Mushtaq et al., 2016; Silvestro et al., 2019). Importantly, detection of miRNAs is fast, uncomplicated, and sensitive, with cost-effective methods allowing their early application as a diagnostic tool (Chen et al., 2008; Gilad et al., 2008; Mitchell and Shiri-Feshki, 2009).

### miRNA a Potential Biomarker for MCI

miRNAs are integral components of biological networks with fundamental roles in regulating gene expression (Singer et al., 2005; Weiner, 2013; Liu et al., 2014b). miRNAs are short non-coding RNA segments, ~20–25-nt in length, that suppress target mRNA translation or induce messenger RNA (mRNA) decay *via* binding with seed sequences in one or more 3'untranslated regions (3'UTR) of target mRNA (Cai et al., 2004; Bartel, 2009; McNeill and van Vactor, 2012). One miRNA can affect many genes involved in the regulation of multiple cellular events and pathways. Circulating miRNA levels have been reported to be transported in blood exosomes, extracellular microvesicles (Kosaka et al., 2010), Argonaute2 (Turchinovich et al., 2011), and other proteins that protect them from being degraded (Geekiyanage et al., 2012).

miRNAs are differentially expressed in specific cell types, organs, and tissues in many human pathologies (Hua et al., 2009). Interestingly, some miRNAs are enriched in specific cellular compartments, such as dendrites and synapses (Lugli et al., 2008; Pichardo-Casas et al., 2012), suggesting a specific role in multiple cellular processes, including development, cell proliferation, replicative senescence, and aging (Qiu et al., 2014; Reddy et al., 2017; Saliminejad et al., 2019). Taking into account that over 70% of reported miRNAs are expressed in the human brain (O'Carroll and Schaefer, 2013; Danka Mohammed et al., 2017) and the crucial role in multiple processes, miRNA could be considered a promising blood biomarker for MCI. Here we performed a systematic review that aims to update and identify potential miRNAs as fluid biomarkers in early stages of AD. Furthermore, we will focus on identifying AD-related target genes modulated by differentially expressed miRNAs in peripheral fluids samples in MCI subjects.

## Methods

**Figure 1:**
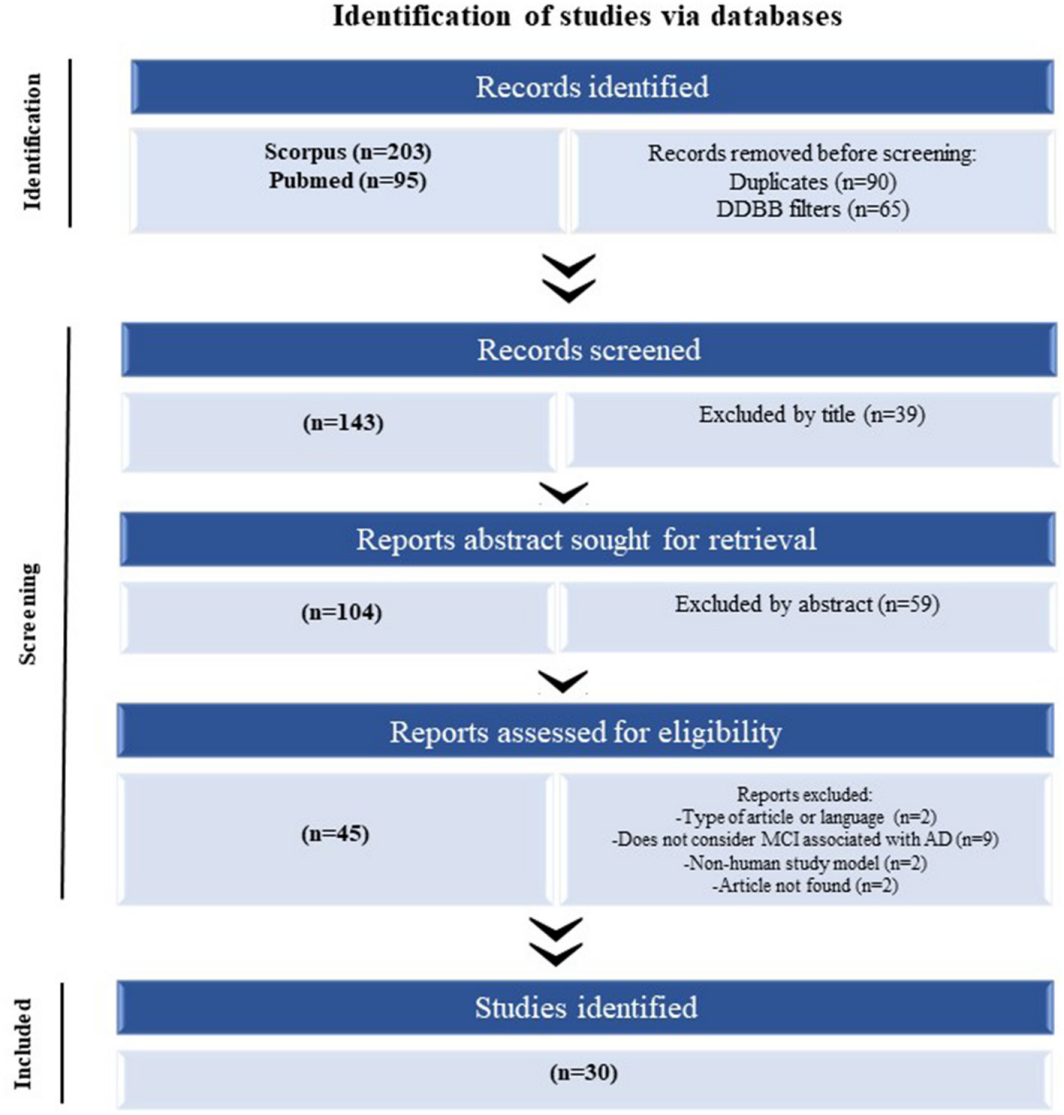
The flow of information of the systematic review according to the PRISMA statement. The search of PubMed and Scopus databases provided a total of 298 citations. Of these, 155 studies were removed after filtering the duplicated and DDBB, and of those, 39 studies were discarded after reviewing the titles. After adjusting for abstracts that did not clearly meet the criteria, 45 articles remained. Finally, the full text of the remaining 45 citations was examined in more detail, and 15 studies did not meet the inclusion criteria as described, achieving a selection of 30 articles for parsing.

A systematic PRISMA review was performed to determine the significantly dysregulated miRNAs in MCI reported in the literature (Page et al., 2021). The search using two search engines (Scopus and PubMed) using the same query was searched on both. For the construction of this query, keywords were defined regarding the source for obtaining the sample (“blood,” “serum,” “plasma,” and “exosome”). The following step was considered in the title, abstract, or author keywords; at least one of the keywords mentioned above, in addition, mentions “miRNA” or “microRNA” and “cognitive impairment.” The last searches were carried out on August 2, 2021 (Figure 1).

The following criteria were considered for the manuscripts to be included in this review: original articles written in English considering miRNA and cognitive impairment related to AD and human samples. Conversely, review, note, book chapter, or any classification other than original article, and articles that do not include cognitive impairment or that are not related to AD were considered an exclusion criterion.

First, the duplicates and manuscripts categorized other than “article” in the search engine were eliminated from the analysis. The next step was to read all abstract articles, eliminating those determined from those that did not meet the inclusion criteria. Finally, articles were read completely, eliminating those that did not meet the inclusion criteria.

In the process of reading the articles included in this review, the following information was extracted: dysregulated miRNAs, change in expression observed, target gene, type of sample studied, the origin of the population, sample size, diagnostic criteria used in the patients evaluated, neuropsychology, age ranges of the evaluated individuals, whether the study is longitudinal or not, miRNA analysis methodology and bioinformatics methodology used.

### The Search Strategy Used in the miRNAs Databases

**Figure 2:**
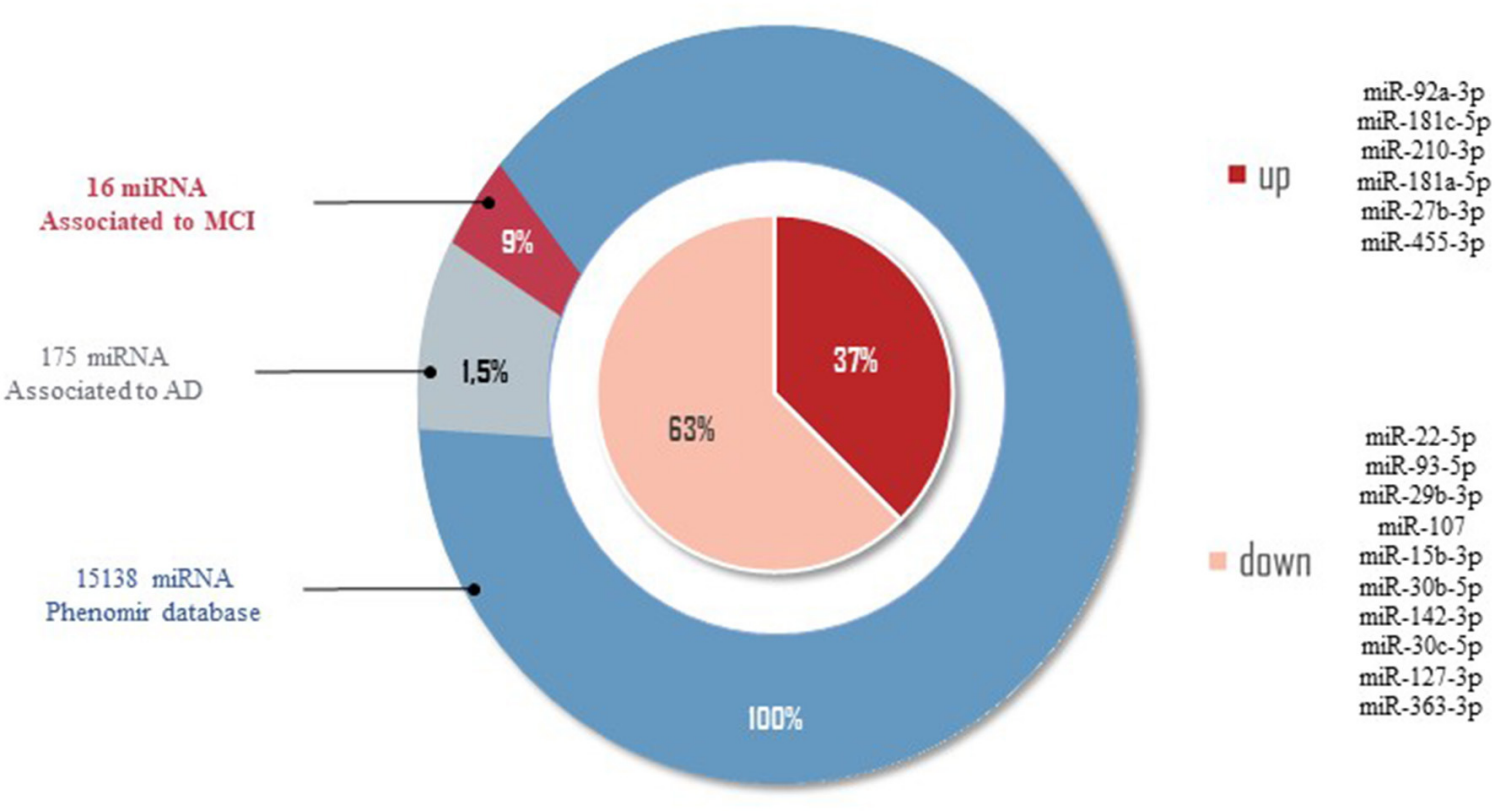
miRNAs related to AD by PhenomiR database. One hundred and seventy-five miRNAs were found after filtering the miRNAs AD-associated. Of those, 10 miRNAs downregulated and six miRNAs upregulated in MCI cases were observed.

**Figure 3:**
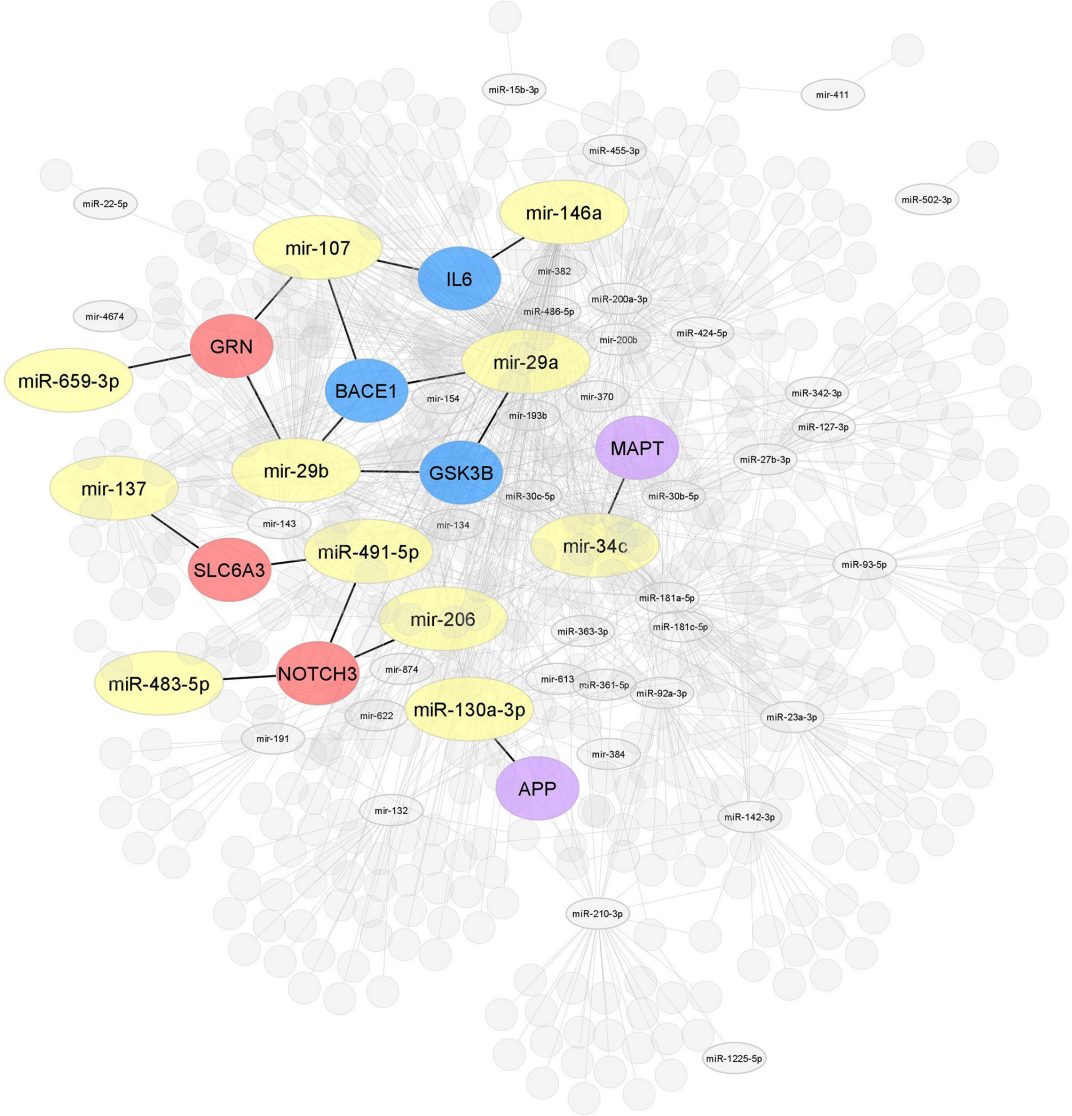
Interaction network of miRNAs-target genes. Interaction network of hub target genes related to AD in blue (*GSK3B, BACE1*, and *IL6*), related to dementia in red (*NOTCH3, SLC6A3*, and *GRN*), and related to both AD and dementia (*APP* and *MAPT*) in purple experimentally validated using several molecular methods. The miRNAs are associated with one or the other of the target genes mentioned above (yellow).

**Figure 4:**
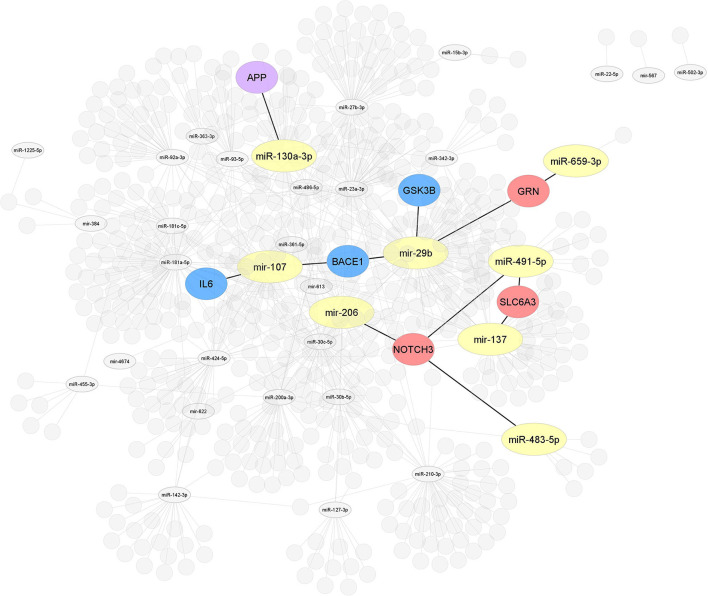
Interaction network of miRNAs-target genes 2. After selecting 86, miRNAs studied in MCI cases were highlighted, all miRNAs (yellow) associated with the modulation of target genes related to AD or other dementias. Interaction network of hub target genes related to AD in blue (*GSK3B, BACE1*, and *IL6*), related to dementia in red (*NOTCH3, SLC6A3*, and *GRN*), and related to both AD and dementia (*APP*) in purple experimentally validated by luciferase reporter assay.

In order to provide a comprehensive overview of differentially regulated miRNA expression data in MCI, we selected miRNAs associated with MCI and AD in the PhenomiR database (Ruepp et al., 2010) (Figure 2). Due to the increasing amount of data in miRNA research, several resources which experimentally validated miRNA targets have been selected, including Tarbase (Papadopoulos et al., 2009), myRTarBase (Hsu et al., 2011) and myRecords (Xiao et al., 2009), and the prediction of miRNA targets by Targetscan (Csardi, 2013) and the miRNA repositories by miRBase (Griffiths-Jones et al., 2006). The miRNAs included addressed the MCI stage, regardless of sex and ethnicity. To perform target interaction, MIENTURNET was used (Licursi et al., 2019) to find the target genes of each miRNA reported in the articles included in this review, using myRTarBase (Hsu et al., 2011). In some articles, miRNAs are generally mentioned without specifying whether it is 3p or 5p; for these purposes, the variation with more reeds between 3p and 5p was considered. Figures 3, 4 were built with Cytoscape (Shannon et al., 2003), which represent two interactomes, the first one being the interactions found for the miRNAs reported in the articles of this review and the second being the subset of these interactions validated by luciferase reporter assays (standard gold method), highlighting the target genes found related to AD or dementia.

## Results

After performing the PRISMA analysis, a total of 30 articles on MCI and miRNAs were identified. The selection process is depicted in the flowchart in Figure 1. We excluded 39 articles by title and 59 articles after reading the abstracts and reviewed the remaining 45 full-text articles, after which a further 15 articles were excluded.

### Demographic and Clinical Features

In chronological order, we systematized the main features of 30 selected articles in Table 1, including the authors and the year of publication of each study, and indicated the study population and sample, clinical criteria, and neuropsychological assessments. Most of the studies came from Asian cohorts (50%), while 33.3% corresponded to European populations, including Italy, Poland, Germany, and others. Only four of them involve cohorts coming from the United States and Canada. Table 1 summarizes the number and means ages of the different study cohorts comprising the selected articles, 13 (43.37%) of them date patients between 63 and 70 years of age, 14 (46.67%) papers describe populations between 70-79 years of age and 3 articles describe patients over 80 years of age (10%). Within the clinical criteria used in the 30 selected articles, 11 (36.6%) of them used the criteria established by the National Institute of Neurological and Communicative Disorders and Stroke and the Alzheimer's Disease and Related Disorders Association (NINCDS-ADRDA), 9 (30%) papers applied the Petersen's criteria. Some authors opted for the Alberts, Winblad, Grundman, IWG-2, DSM-IV criteria to a lesser extent. Among all reported neuropsychological assessments for MCI subjects, 16 (53.3%) studies used MMSE scores of 24 or higher, while 8 (26.67%) articles used MoCA scores ranging from 17 to 23. Table 1 notes that 9 (30%) articles have applied a clinical criterion or a neuropsychological tool as inclusion criteria for MCI.

**Table 1:** Articles resume table.

**References**	**DOI**	**Country**	**Sample**	**Sample size (*n*)**	**Age, years**	**Clinical criteria**	**Neuropsychological assessments for MCI**
Geekiyanage et al. (2012)	10.1016/j.expneurol.2011.11.026	United Kingdom	Serum	MCI (3), early MCI (1), AD (7), early AD (3), HC (7)	MCI (86 ± 1), early MCI (89), AD (88 ± 6.5), early AD (89 ± 2.5), HC (86.8 ± 3.5)	NA	MMSE
Sheinerman et al. (2013)	10.18632/aging.100624	California, United States	Plasma	MCI (50), age-matched controls (50)	MCI (68.2 ± 10.5), age-matched controls (65.1 ± 10.8)	NA	ADAS-Cog, CDRS, MMSE, Wechsler Memory Scale
Liu et al. (2014c)	10.1016/j.brainres.2014.04.026	Xuanwu, China	Blood	MCI (31), AD (38), HC (30)	MCI (72.8 ± 6.1), AD (76.8 ± 6.8), HC (75.2 ± 6.5)	NA	NA
Liu et al. (2014b)	10.3892/ijmm.2014.1780	Beijin, China	Serum	MCI (32), AD (45), HC (50)	MCI (63.2 ± 6.1), AD (64.2 ± 5.8), HC (63.9 ± 5.7)		NA
Liu et al. (2014a)	10.3892/mmr.2014.2484	Beijin, China	Plasma exosome	MCI (43), AD (51)	MCI (63.8 ± 6.1), AD (64.2 ± 6.5)	NA	NA
Dong et al. (2015)	10.1155/2015/625659	Shangai, China	Serum	MCI (30), AD (127), HC (123)	MCI (81.1 ± 6.8), AD (79.3 ± 8.9), HC (79.5 ± 6.8)	NA	MMSE
Zhu et al. (2015)	10.3892/etm.2015.2179	Zaozhuang, China	Serum	MCI (30), AD (26), HC (42)	MCI (71.6 ± 7.8), AD (72.3 ± 8.1), HC (71.9 ± 7.8)	NINCDS-ADRDA	NA
Li et al. (2016)	10.5582/bst.2016.01127	Tianjin, China	Serum	MCI (42), AD (48), HC (40)	MCI (64.8 ± 7.2), AD (65.5 ± 6.8), HC (63.2 ± 6.3)	NINCDS-ADRDA	NA
Guedes et al. (2016)	10.1016/j.dadm.2015.11.004	Portugal	Blood-derived monocytes and monocyte-derived macrophages	MCI (52), AD (36), age-matched HC (36)	MCI (73.6 ± 8.9), AD (74.9 ± 8.8), amHC (70.3 ± 6.6)	NINCDS-ADRDA, Albert's and Petersen's criteria	MMSE, MoCA, the Alzheimer's Disease Assessment Scale-Cognitive (ADAS-Cog), Battery of Lisbon for the Assessment of Dementia and the clinical dementia rating (CDR), and the geriatric depression scale (GDS-30) to exclude depression.
Kayano et al. (2016)	10.1186/s40364-016-0076-1	Japan	Plasma	MCI (23), HC (30)	MCI (72.8 ± 3.1), HC (70.4 ± 4.9)	Petersen criteria	MMSE
Keller et al. (2016)	10.1016/j.jalz.2015.12.012	German/ United States	Blood	MCI (20), AD (103), HC (77)	MCI (67.7 ± 8.2), AD (70.7 ± 8.2), HC (67.3 ± 7.8)	NA	MMSE, MoCA
Wang et al. (2016)	10.3389/fnagi.2016.00112	China	Plasma	aMCI (20), cognitively normal HC (24)	aMCI (70.1 ± 7.2), cnHC (69.9 ± 7.6)	Medical history, physical and neurological examinations, and MRI scans.	MMSE, MoCA
Zirnheld et al. (2016)	10.4172/0975-9042.000117	Montreal, Canada	Plasma	MCI (34), Mild AD (16), Moderate-Severe AD (20), normal elderly controls (NEC, 37)	MCI (75.3 ± 5.4), Mild AD (78.2 ± 3.5), Moderate-Severe AD (77.7 ± 6.8), NEC (75.3 ± 5.4)	Winblad's criteria	Full neuropsychological assessment including Modified WAIS subtests (digit symbol), Wechsler Memory Scale-R subtests (Logical Memory, Digit Span), Raven's Progressive Matrices, Visual perceptual tests, RAVLT, as well as Verbal Fluency, MoCA and MMSE.
Kumar et al. (2017)	10.1093/hmg/ddx267	Texas, United States	serum/plasma	MCI (20), AD (11), HC (18)	MCI (74 ± 11.1), AD (73 ± 13.5), HC(77 ± 17.5)	NINCDS-ADRDA	MMSE
Nagaraj et al. (2017)	10.18632/oncotarget.15109	Poland	Plasma	MCI-AD (15), AD (20), age-matched HC (15)	MCI-AD (65.8 ± 7), AD (67.5 ± 8), age-matched HC (66 ± 3)	DSM-IV, NINCDS-ADRDA and Alzheimer's Association workgroups on diagnostic guidelines for Alzheimer's disease	MMSE
Yang et al. (2018)	10.3967/bes2018.011	China	Serum exosomal	MCI (101), AD (107)	MCI (61.63 ± 7.32), AD (74.15 ± 7.93)	NINCDS-ADRDA	NA
Agostini et al. (2019)	10.3389/fnagi.2019.00052	Italy	Blood	MCI (22), AD (22), HC (22)	MCI (76 ± 4.0), AD (78 ± 4.0), HC (70 ± 9.9)	NINCDS-ADRDA, Petersen and Grundman criteria, MRI, computed tomography scan.	MMSE
Lu et al. (2019)	10.1007/s12264-019-00361-0	Tongji, China	Blood	aMCI (10), AD (7), HC (17)	aMCI (70.20 ± 10.40), AD (70.43 ± 5.08), HC (70.86 ± 4.11)	NINCDS-ADRDA, exclusion of brain tumor, vascular dementia, and cerebrovascular diseases	MMSE
Salama et al. (2019)	10.3889/oamjms.2019.834	Egypt	Plasma	adults (186), MCI was detected among 14	Between 40 and 65 years, mean 51.3 ± 4.1 years	National Institute on Aging–Alzheimer's Association recommendations	ACE III, MoCA, Quick cognitive tests (Quick MCI)
Siedlecki-Wullich et al. (2019)	10.1186/s13195-019-0501-5	Barcelona, Spain	Plasma	MCI (26), AD (56), HC (38)	MCI (72.0 ± 8.49), AD (77.77 ± 6.69), HC (68.29 ± 8.99)	Petersen criteria	Neuropsychological battery used in Fundació ACE (NBACE) and tests
Zhao et al. (2020)	10.1373/jalm.2019.029595	England	Serum	MCI (13), AD (51), HC (32)	MCI (75 ± 11), AD (73 ± 5), HC (80 ± 10)	NINCDS-ADRDA and Petersen criteria.	NA
de Felice et al. (2020)	10.1007/s12035-020-02029-7		Blood/Serum	MCI-AD (18), AD (18)	MCI-AD (69.9 ± 5.04), AD (70.8 ± 10.22)	Petersen and IWG-2 criteria	Full neuropsychological assessment and MMSE
Li et al. (2020)	10.1016/j.neuroscience.2020.02.044	Jinzhou, China	Plasma-derived Extracellular Vesicles	MCI (28), AD (31), HC (28)	MCI (76.6 ± 5.4), AD (74.2 ± 6.1), HC (74.6 ± 5.0)	NINCDS–ADRDA	NA
Al-Rawaf et al. (2021)	10.2147/CIA.S285689	Saudi Arabia	Serum	MCI (70), HC (80)	MCI (64.9 ± 4.1), HC (65.3 ± 3.5)	Petersen criteria	The Clinical Dementia Rating scale, MoCA, MMSE
Liu et al. (2021)	10.1155/2021/5450397	Xuanwu and Beijing, China	Blood, Serum, Plasma exosomes	subjective cognitive decline (SCD, 89), MCI (92), AD (92), HC (60)	SCD (75.7 ± 4.9), MCI (75.2 ± 8.0), AD (78.1 ± 7.2), HC (76.5 ± 6.1)	NA	NA
He et al. (2021)	10.3233/JAD-210307	China	Plasma	MCI (5,20,40), NC (5,10,40)	MCI (68 ± 2.83~78.25 ± 5.48)/NC(70 ± 2.12~78.23 ± 5.62)	Petersen criteria	Full neuropsychological assessment including: MMSE, MoCA, Wechsler Memory Scale, Verbal Fluency Test-fruits and idioms, Hopkins Verbal Learning and 30-min Delayed Test, Visual Recognition Function Test.
*Sheinerman et al. (2012)	10.18632/aging.100486	California, United States	Plasma	MCI (30), AD (30), age matched HC (30)	MCI (81.7 ± 3.2), AD (76.9 ± 3.5) age matched HC (77.4 ± 4.9)	NINCDS–ADRDA, Petersen criteria	Consensus team determined cognitive status after completing the Consortium to Establish a Registry for Alzheimer's Disease (CERAD) battery
*Xie et al. (2017)	10.3233/JAD-160468	China	Serum	aMCI-aMCI (330), aMCI-AD (128)	aMCI-aMCI (69.91 ± 4.01), aMCI-AD (76.04 ± 4.82)	Petersen criteria	Wechsler Memory Scale Revised (WMS-R, Chinese version), Clinical Dementia Rating (CDR), MMSE and MoCA
*Ansari et al. (2019)	10.1016/j.neurobiolaging.2019.06.005	European Alzheimer's Disease Neuroimaging Initiative	Blood	progressor MCI (19), stable MCI (26)	pMCI (66.68 ± 5.8), sMCI (66.23 ± 7.1)	NA	MMSE, CDR, logical memory test. They also used the geriatric depression scale (GDS-15) to exclude depression.
*Kenny et al. (2019)	10.3390/biom9110734	Madrid, Spain	Plasma	MCI (30), AD (25), HC (31)	MCI (76.8 ± 4.0), AD (84.6 ± 3.5), HC (75.0 ± 4.7)	NA	Full neuropsychological assessment including: MMSE, Free and Cued Selective Reminding Test (FCSRT), Clinical Dementia Rating (CDR) and Functional Activities Questionnaire (FAQ)

*Selected articles used for the data analysis show an organization following an order by authors, year, digital object identifier (DOI), country, sample, sample size (n), age, clinical criteria, and neuropsychological assessment for MCI*.

* *Longitudinal studies*.

### miRNAs Identified in MCI Subjects

The miRNAs reported in the 30 selected articles were analyzed in three different stages. In the first stage, the discovery stage of miRNA families MCI-associated. In the second stage, miRNAs were detected as differentially expressed between MCI and AD in longitudinal studies. Finally, in the third stage, the detected miRNAs and their target genes are based on the information reported in databases.

#### Stage 1: miRNAs Family Reported in MCI Cases

Members from the same miRNA family are important because they have a typical sequence in genes that hints a shared function (Kaczkowski et al., 2009). miRNA genes in a family exhibit full or partial conservation of miRNAs seed sequences (Mathelier and Carbone, 2013). Notably, more evidence suggests that miRNA genes in the same miRNA family are non-randomly co-localized around genes involved in neurodegenerative diseases, cancer, immune system, among others (Kamanu et al., 2013). Of the 86 miRNAs reported in 30 selected articles, we have identified 4 miRNAs' families; 2 downregulated miRNA families (mir-29 and mir-30), 1 upregulated miRNA family (mir-200), and 1 up-/downregulated miRNA family (mir-181) (see Table 2A).

**Table 2:** Differentially expressed peripheral fluid-associated miRNAs in MCI.

		**Sample**					
**miRNA**	**Up/down**	**Blood**	**Plasma**	**Serum**	**Plasma/** **serum exosome**	**Target genes**	**Publication**
**(A) miRNA FAMILIES**							
**mir-29**							
mir-29a	Down			X		*SPTLC1/2*	Geekiyanage et al., 2012
mir-29b	Down			X		*SPTLC1/2*	Geekiyanage et al., 2012
miR-29b-3p	Down		X			*BACE 1*	He et al., 2021
**mir-30**							
miR-30b-5p	Down		X			*PSEN2*	Nagaraj et al., 2017
miR-30c-5p	Down	X				*-*	Keller et al., 2016
**mir-181**							
mir-181a	Up	X	X			*Fidgetin, B-cell lymphoma 2, SIRT1*	Sheinerman et al., 2013; Ansari et al., 2019
miR-181a-5p	Up	X				*SNAP-25*	Agostini et al., 2019
mir-181c	Down		X	X		*SPTLC1/2*	Geekiyanage et al., 2012; Zirnheld et al., 2016
miR-181c-5p	Up		X			*NPTX1, NPTXR*	Siedlecki-Wullich et al., 2019
**mir-200**							
miR-200a-3p	Up		X			*MAPK, BACE1*	Nagaraj et al., 2017
mir-200b	Up	X				*APP, Aβ42, MGAT3*	Liu et al., 2014a; Guedes et al., 2016
**(B) miRNA REPORTED IN LONGITUDINAL STUDIES**							
mir-9	Down		X	X		*SPTLC1/2*	Geekiyanage et al., 2012; Sheinerman et al., 2013
mir-29a	Down			X		*SPTLC1/2*	Geekiyanage et al., 2012
mir-29b	Down			X		*SPTLC1/2*	Geekiyanage et al., 2012
miR-30b-5p	Down		X			*-*	Nagaraj et al., 2017
mir-34c	Down		X			*SIRT1, ONECUT2, BCL2*	Zirnheld et al., 2016
mir-137	Down			X		*SPTLC1/2*	Geekiyanage et al., 2012
miR-142-3p	Down		X	X		*BDNF, DPP4, SIRT1, FRS2, PP2A, PI3K*	Nagaraj et al., 2017; Al-Rawaf et al., 2021
mir-146a	Up	X		X		*CFH, TLR2*	Dong et al., 2015; Ansari et al., 2019
mir-181a	Up	X	X			*SIRT1*	Sheinerman et al., 2013; Ansari et al., 2019
mir-181c	Down		X	X		*SPTLC1/2*	Geekiyanage et al., 2012; Zirnheld et al., 2016
miR-200a-3p	Up		X			*MAPK, BACE1*	Nagaraj et al., 2017
mir-206	Up		X	X		*BDNF, SIRT1*	Xie et al., 2017; Kenny et al., 2019
mir-411	Down		X			*-*	Zirnheld et al., 2016
miR-455-3p	Up		X	X		*TGF-β1, APP*	Kumar et al., 2017
miR-483-5p	Up		X	X		*BDNF, DPP4, SIRT1, BACE1, MAPT*	Nagaraj et al., 2017; Al-Rawaf et al., 2021
miR-486-5p	Up		X			*BACE1, IGFRI, MAPT*	Nagaraj et al., 2017
miR-502-3p	Up		X			*SSTR, SOD2*	Nagaraj et al., 2017
mir-567	Down	X		X		*NEUROD2, NEUROG2, TCF3, TCF4, TBR1*	de Felice et al., 2020
mir-613	Up			X		*BDNF*	Li et al., 2016
mir-3658	Up	X		X		*-*	de Felice et al., 2020
mir-3908	Up	X		X		*-*	de Felice et al., 2020
miR-5588-5p	Up	X		X		*-*	de Felice et al., 2020
**(C) OTHERS miRNA MCI-ASSOCIATED**							
mir-7	Up/down		X			*-*	Sheinerman et al., 2013
miR-15b-3p	Down	X			X	*SNAP-25*	Agostini et al., 2019; Li et al., 2020
miR-22-5p	Down		X			*BACE 1*	He et al., 2021
mir-23a	Up	X				*CCR2*	Guedes et al., 2016
miR-23a-3p	Up	X				*SNAP-25*	Agostini et al., 2019
miR-27b-3p	Up	X				*SNAP-25*	Agostini et al., 2019
miR-92a-3p	Up		X			*NPTX1, NPTXR*	Siedlecki-Wullich et al., 2019
mir-93	Up			X		*CFH*	Dong et al., 2015
miR-93-5p	Down				X	*MAPT*	Li et al., 2020
mir-107	Down		X			*BACE 1*	Wang et al., 2016
mir-124a	Up/down			X		*BDNF, DPP4, SIRT1*	Al-Rawaf et al., 2021
mir-125b	Up/down		X	X		*BDNF, DPP4, SIRT1, TP53*	Sheinerman et al., 2013; Kayano et al., 2016; Al-Rawaf et al., 2021
miR-127-3p	Down	X				*-*	Sheinerman et al., 2013
mir-128	Up	X	X			*CCR2*	Sheinerman et al., 2013; Guedes et al., 2016
miR-130a-3p	Up	X				*SNAP-25*	Agostini et al., 2019
mir-132	Up		X			*Rasa1, BDNF, SIRT1*	Sheinerman et al., 2013; Salama et al., 2019
mir-134	Up		X			*-*	Sheinerman et al., 2013; Salama et al., 2019
miR-134-3p	Down		X			*BACE 1*	He et al., 2021
mir-135	Down or undetectable		X			*-*	Kenny et al., 2019
miR-135a	Down				X	*BACE1, APP*	Yang et al., 2018
mir-138	Up	X				*APP*	Lu et al., 2019
mir-143	Down			X		*CFH*	Dong et al., 2015
mir-154	Up	X				*CCR2*	Guedes et al., 2016
mir-191	Up		X			*TP53*	Kayano et al., 2016
mir-193b	Up/down	X		X	X	*BACE1, APP*	Liu et al., 2014a; Yang et al., 2018
mir-210	Down			X		*VEGF*	Zhu et al., 2015
miR-210-3p	Up		X			*NPTX1, NPTXR*	Siedlecki-Wullich et al., 2019
miR-323-3p	Up/down		X			*-*	Sheinerman et al., 2013
miR-342-3p	Down				X	*-*	Li et al., 2020
miR-361-5p	Down	X				*SNAP-25*	Agostini et al., 2019
miR-363-3p	Down	X				*-*	Keller et al., 2016
mir-370	Down		X			*-*	Sheinerman et al., 2013
mir-382	Up/down		X			*-*	Sheinerman et al., 2013
mir-384	Down			X	X	*BACE 1, APP*	Liu et al., 2014c; Yang et al., 2018
miR-424-5p	Down				X	*MAPT*	Li et al., 2020
miR-491-5p	Down		X			*-*	Sheinerman et al., 2013
mir-554	Down		X			*BACE 1*	He et al., 2021
mir-604	Down		X			*BACE 1*	He et al., 2021
mir-622	Down		X			*BACE 1*	He et al., 2021
miR-659-3p	Down		X			*BACE 1*	He et al., 2021
mir-874	Up/down		X			*-*	Sheinerman et al., 2013
miR-1185-2-3p	Down		X			*BACE 1*	He et al., 2021
miR-1225-5p	Down		X			*BACE 1*	He et al., 2021
miR-1306-5p	Up				X	*MAPT*	Li et al., 2020
miR-1909-3p	Down		X			*BACE 1*	He et al., 2021
mir-1972	Down		X	X		*TGF-β1, APP*	Kumar et al., 2017
miR-3065-5p	Down				X	*MAPT*	Li et al., 2020
miR-3124-3p	Up		X	X		*TGF-β1, APP*	Kumar et al., 2017
miR-3156-5p	Down		X			*BACE 1*	He et al., 2021
miR-3613-3p	Up		X	X		*TGF-β1, APP*	Kumar et al., 2017
mir-4317	Up		X	X		*TGF-β1, APP*	Kumar et al., 2017
miR-4659b-5p	Down		X			*BACE 1*	He et al., 2021
miR-4668-5p	Up		X	X		*TGF-β1, APP*	Kumar et al., 2017
mir-4674	Up		X	X		*TGF-β1, APP*	Kumar et al., 2017
mir-4698	Down		X			*BACE 1*	He et al., 2021
mir-5691	Down		X			*BACE 1*	He et al., 2021
mir-6722	Down		X	X		*TGF-β1, APP*	Kumar et al., 2017
miR-6856-3p	Up		X	X		*TGF-β1, APP*	Kumar et al., 2017
Let-7b	Up		X			*-*	Kenny et al., 2019

*(A) miRNAs families up/downregulated in MCI cases*.

*(B) miRNAs up/downregulated revealed in four longitudinal studies*.

*(C) Other miRNAs mentioned in the 30 articles MCI-associated. miRNAs articles used for the data analysis, showing an organization following an order by type of miRNA, up/down expression level, sample (blood, plasma, serum, plasma/serum exosome), altered gene expression, and publication*.

Five miRNAs have been shown to be downregulated in serum and plasma samples from MCI cases in Asian and European cohorts. Three of them belong to 2 isomiR (mir-29a and mir-29b) of the mir-29 family (Geekiyanage et al., 2012). The other two miRNA sequences come from 2 isomiR sequences (mir-30b and mir-30c) belonging to the mir-30 family (Keller et al., 2016; Nagaraj et al., 2017). On the other hand, of the 30 articles, only three of them have reported 1 upregulated miRNA family. miR-200a-3p sequence, a product of mir-200a, showed upregulation in blood samples from MCI subjects in a Polish MCI cohort (Liu et al., 2014a; Guedes et al., 2016; Nagaraj et al., 2017). Six selected articles have reported 4 miRNAs to belong to 1 miRNAs family which shows to be up- or downregulated depending on the type of isomiR, mature sequences, and precursor-miRNA. miR-181a-5p, miR-181c-5p, and mir-181a sequences were upregulated in plasma samples in European MCI populations (Nagaraj et al., 2017; Agostini et al., 2019; Ansari et al., 2019). However, two studies reported that mir-181c isomiR levels in serum and plasma samples in North America MCI subjects were downregulated (Geekiyanage et al., 2012; Zirnheld et al., 2016).

Often genes in the same miRNA family exhibit similar functions due to their structural configuration, suggesting that miRNA genes could be involved in a familiar regulatory role (Kaczkowski et al., 2009). Our results showed that of the 4 miRNA families found, one of them (mir-181) reported several related members in both up- and downregulated.

#### Stage 2: miRNAs Reported in Longitudinal Studies

Longitudinal studies allow the study of changes that occur in the progression of the disease and aim to make predictions of the development of AD (Sheinerman et al., 2013; Xie et al., 2017; Ansari et al., 2019). Four articles were found that correspond to longitudinal studies where the expression of miRNAs in stages of MCI and its progression to AD was assessed. The average follow-up was between 1 and 5 years (see Table 2B).

In a first longitudinal study performed in MCI subjects, two miRNAs, mir-132 and mir-134, were evaluated (Sheinerman et al., 2013). The study was performed in 19 healthy control (HC) subjects with a follow-up of 2–5 years where, after the follow-up, nine subjects continued as HC and 10 subjects progressed to MCI. Regarding the results identified for mir-132, which considers miRNAs mir-128, mir-132, mir-874, and miR-491-5p, significant differences are observed between MCI and HC, with a 79–89% sensitivity and a specificity of 83–100% in discriminating subjects converting from a normal cognitive state to MCI. On the other hand, the mir-134, which considers miRNAs mir-134, miR-323-3p, mir-382, and mir-370, can discriminate subjects converting from a normal state to MCI with a sensitivity of 80–95% and a specificity of 79–84% (Sheinerman et al., 2013).

In 2017, another study evaluated a larger cohort of 458 subjects with MCI, followed up for 5 years, classifying subjects into amnestic mild cognitive impairment (aMCI) stable (*n* = 330) and MCI-converted AD (*n* = 128). Interestingly, the expression level of mir-206 was increased in MCI subjects who progressed to AD compared to subjects who remained stable MCI (Xie et al., 2017). By ROC (Receiver Operating Characteristic) analysis, an AUC (Area Under the ROC Curve) of 0.95 was described, with a sensitivity of 95.3% and a specificity of 77.8% for identifying MCI subjects who progressed to AD, concluding that increased serum mir-206 levels would accelerate the progression of aMCI to AD (Xie et al., 2017).

Additionally, another longitudinal study was performed with a 4-year follow-up of a small cohort classified into three groups, group 1 (*n* = 6) stable MCI, group 2 (*n* = 6) controls converting to AD, and group 3 (*n* = 6) subjects with MCI converting to AD (Kenny et al., 2019). Likewise, Xie et al. showed that the levels mir-206 were increased in MCI and MCI converting to AD (Xie et al., 2017).

Subsequently, subjects were classified depending on the results in two cognitive tests, Free and Cued Selective Reminding Test (FCSRT) and MMSE, where it was identified that higher levels of mir-206 expression correlate with more significant memory deficit (FCSRT) with an AUC of 0.82 and more significant cognitive impairment measured by MMSE with an AUC of 0.83 (Kenny et al., 2019).

Finally, the last longitudinal study identified in this systematic review was conducted with a 2-year follow-up of 44 subjects with MCI, where 25 subjects remained stable MCI while 19 subjects converted to AD (Ansari et al., 2019). Two miRNAs, including mir-146a and mir-181a, were evaluated in this study, showing increased expression in MCI subjects that converted to AD compared to stable MCI (Ansari et al., 2019). In the same study, correlations were found between increased expression of these miRNAs and Aβ CSF levels, hippocampal atrophy, and alterations in l regions (Ansari et al., 2019).

#### Stage 3: Database of miRNA Related to MCI and AD

Of the 2633 miRNAs structural characterized in the miRBase databases (Griffiths-Jones et al., 2006, 2008), one-third of these miRNAs are found in the coding part of genes, and the remaining are in the intronic regions, meaning that 33% of miRNAs modulate different target genes, being of our interest those miRNAs modulating AD-related target genes. In order to provide an overview of the differentially expressed data of miRNAs in MCI, we selected from the 86 miRNAs those that correlate with AD by the PhenomiR database (Ruepp et al., 2010). Of the 15138 different miRNAs associated with several diseases, 175 miRNAs (1.5%) were associated with AD. Of these 175 miRNAs, only 16 miRNAs (9%) dysregulated in MCI cases were observed (see Figure 2). Thirty-seven percent of miRNAs associated in both AD and MCI have shown to be upregulated, including miR-27b-3p, miR-92a-3p, miR-181c-5p, miR-181a-5p, miR-210-3p, miR-455-3p, while 67% of miRNAs were downregulated, including miR-15b-3p, miR-22-5p, miR-29b-3p, miR-30c-5p, miR-30b-5p, miR-93-5p, miR-107, miR-127-3p, miR-142-3p, and miR-363-3p (Figure 2). This result suggests that these 16 miRNAs could be potential prognostic biomarkers for MCI.

In order to provide a comprehensive search of the targets of the 86 miRNAs obtained from our systematic review, two approaches were used. First, we noted all putative target genes reported in 30 articles by several methods (see Table 2). Over half of those target genes were validated by qRT-PCR, one-third by sequencing, followed by other techniques such as western blotting, miRNA microarrays, and *in situ* hybridization. Second, we highlight the miRNAs associated with target genes validated by the well-known standard gold method luciferase reporter assay (see Table 3).

**Table 3:** Validate target genes regulated by peripheral fluid-associated miRNAs in MCI cases.

**miRNA**	**Up/down**	**Targets genes reported in 30 articles**	**Validated targets by several methods**	**Validated targets by luciferase reporter assays**	**Publication**
miR-15b-3p	Down	*SNAP-25*	295		Agostini et al., 2019; Li et al., 2020
miR-22-5p	Down	*BACE 1*	1,135		He et al., 2021
miR-23a-3p	Up	*SNAP-25*	2,093		Agostini et al., 2019
miR-27b-3p	Up	*SNAP-25*	2,761		Agostini et al., 2019
miR-29b-3p	Down	*BACE 1*	1,781	*GSK3B, BACE1, GRN*	He et al., 2021
miR-30b-5p	Down	*PSEN2*	2,139		Nagaraj et al., 2017
miR-30c-5p	Down	*-*	2,377		Keller et al., 2016
miR-92a-3p	Up	*NPTX1, NPTXR*	3,344	*SIRT1*	Siedlecki-Wullich et al., 2019
miR-93-5p	Down	*MAPT*	4,119		Li et al., 2020
mir-107	Down	*BACE 1*	4,403	*IL6, BACE1*	Wang et al., 2016
miR-127-3p	Down	*-*	108		Sheinerman et al., 2013
miR-130a-3p	Up	*SNAP-25*	2,704	*APP*	Agostini et al., 2019
miR-134-3p	Down	*BACE 1*	89		He et al., 2021
mir-137	Down	*SPTLC1/2*	985	*NOTCH3, SLC6A3*	Geekiyanage et al., 2012
miR-142-3p	Down	*BDNF, DPP4, SIRT1, FRS2, PP2A, PI3K*	1416		Nagaraj et al., 2017; Al-Rawaf et al., 2021
miR-181a-5p	Up	*SNAP-25*	2,933	*SIRT1*	Agostini et al., 2019
miR-181c-5p	Up	*NPTX1, NPTXR*	1,504		Siedlecki-Wullich et al., 2019
miR-200a-3p	Up	*MAPK, BACE1*	900	*TRAPPC8*	Nagaraj et al., 2017
mir-206	Up	*BDNF, SIRT1*	234	*NOTCH3, G9PD, GPD2, PGD, TKT*	Xie et al., 2017; Kenny et al., 2019
miR-210-3p	Up	*NPTX1, NPTXR*	3,624	*BDNF, NPTX1*	Siedlecki-Wullich et al., 2019
miR-342-3p	Down	*-*	1,019		Li et al., 2020
miR-361-5p	Down	*SNAP-25*	1,154		Agostini et al., 2019
miR-363-3p	Down	*-*	937		Keller et al., 2016
mir-384	Down	*BACE 1, APP*	131		Liu et al., 2014a; Yang et al., 2018
miR-424-5p	Down	*MAPT*	3,021		Li et al., 2020
miR-455-3p	Up	*TGF-β1, APP*	1,526		Kumar et al., 2017
miR-483-5p	Up	*BDNF, DPP4, SIRT1, BACE1, MAPT*	388		Nagaraj et al., 2017; Al-Rawaf et al., 2021
miR-486-5p	Up	*BACE1, IGFRI, MAPT*	372		Nagaraj et al., 2017
miR-491-5p	Down	*-*	637	*NOTCH3, SLC6A3*	Sheinerman et al., 2013
miR-502-3p	Up	*dynactin, SSTR, Crk, SOD2*	532		Nagaraj et al., 2017
mir-554	Down	*BACE 1*	36		He et al., 2021
mir-567	Down	*NEUROD2, NEUROG2, TCF3, TCF4, TBR1*	356		de Felice et al., 2020
mir-604	Down	*BACE 1*	109		He et al., 2021
mir-613	Up	*BDNF*	129	*BDNF*	Li et al., 2016
mir-622	Down	*BACE 1*	265		He et al., 2021
miR-659-3p	Down	*BACE 1*	159	*GRN*	He et al., 2021
miR-1185-2-3p	Down	*BACE 1*	194		He et al., 2021
miR-1225-5p	Down	*BACE 1*	179		He et al., 2021
miR-1306-5p	Up	*MAPT*	655		Li et al., 2020
miR-1909-3p	Down	*BACE 1*	260		He et al., 2021
mir-1972	Down	*TGF-β1, APP*	154		Kumar et al., 2017
miR-3065-5p	Down	*MAPT*	443		Li et al., 2020
miR-3124-3p	Up	*TGF-β1, APP*	231		Kumar et al., 2017
miR-3156-5p	Down	*BACE 1*	84		He et al., 2021
miR-3613-3p	Up	*TGF-β1, APP*	914		Kumar et al., 2017
mir-3658	Up	*-*	222		de Felice et al., 2020
mir-3908	Up	*-*	124		de Felice et al., 2020
mir-4317	Up	*TGF-β1, APP*	120		Kumar et al., 2017
miR-4659b-5p	Down	*BACE 1*	111		He et al., 2021
miR-4668-5p	Up	*TGF-β1, APP*	548		Kumar et al., 2017
mir-4674	Up	*TGF-β1, APP*	65		Kumar et al., 2017
mir-4698	Down	*BACE 1*	402		He et al., 2021
miR-5588-5p	Up	*-*	67		de Felice et al., 2020
mir-5691	Down	*BACE 1*	166		He et al., 2021
miR-6856-3p	Up	*TGF-β1, APP*	142		Kumar et al., 2017

##### Target Genes Validated by qRT-PCR/Sequencing/Others

Most of the reported miRNAs regulate the expression of AD-related genes, of which 21 miRNAs regulate the β-site amyloid-protein precursor (*APP*) cleaving enzyme 1 (*BACE1*) and 13 miRNAs regulate. Although *APP*- and *BACE1*-associated miRNAs suggest that the regulatory process is complex and extensive, 85% of *BACE1*-associated miRNAs and 62% of APP-associated miRNAs were observed to be downregulated. Among the 30 selected articles, other target genes associated with the 86 miRNAs mentioned were a brain-derived neurotrophic factor (*BDNF*), Sirtuin 1 (*SIRT1*), transforming growth factor-beta receptor 1 (*TGF-*β*1*), microtubule-associated protein tau (*MAPT*), serine palmitoyltransferase long chain base subunit 1 and subunit 2 (*SPTLC1/2*), synaptosome associated protein 25 (*SNAP-25*), and dipeptidyl peptidase 4 (*DPP4*) genes.

Interactome 1 shows a schematic representation of the target genes of the 86 miRNAs AD-associated, other dementias, and both (AD and other dementias) (Figure 3). The mir-107, miR-29b-3p, miR-29a-3p, miR-146a-5p showed a direct interaction with the gene targets *BACE1, IL6*, and *GSK3B*; three target genes highly AD-associated. The mir-107, miR-29b-3p, miR-659-3p, mir-137, miR-491-5p, miR-483-5p, and mir-206 presented a direct association with the targets *GRN, SLC6A3*, and *NOTCH3*; genes related to other dementias. Finally, miR-34c-5p and miR-130a-3p reflected an interaction with the *MAPT* and *APP* gene, both in dementia and AD-associated (Figure 3).

##### Target Genes Validated by Luciferase Reporter Assay

Of the 86 miRNAs evaluated, 55 were further analyzed, 31 miRNAs were discarded from this analysis because they were not recognized in the evaluated databases. Table 3 shows the total output of validated gene targets for each of the 55 miRNAs. Within this search, mir-554 showed the lowest count of targets with 36 validated ones, while the mir-107 presented the highest count with 4,403 targets (Table 3). Due to the large volume of data, we selected only those targets validated by luciferase reported assay. Figure 4 shows the interaction between the 55 miRNAs and the target genes AD and other dementias associated. The mir-107 and miR-29b-3p showed a direct interaction with the AD-associated gene targets. The miR-29b-3p, miR-659-3p, mir-137, miR-491-5p, miR-483-5p, and mir-206 presented a direct association with the targets related to other dementias, while miR-130a-3p reflected an interaction with *APP* gene, both in dementia and AD-associated (Figure 4).

Our results claim that of the total 86 miRNAs MCI-associated, only the mir-107 and miR-29b-3p were reported by the whole database analyzed. In addition, those miRNAs also showed close interaction with AD-related target genes, including *BACE 1, IL6*, and *GSK3B* (Figures 3, 4). An interesting insight derived from our analysis is (i) the first one, the miR-29a-3p, miR-146a-5p, miR-34c-5p, and miR-130a-3p, were related to AD-associated genes, but not characterized in the PhenomiR database as miRNAs AD-associated (Figure 3), being the interest as potential prognostic biomarkers for early-stage AD and (ii) although the mir-181 family did not show a direct interaction with AD-related genes, several members were up-and downregulated in cases of MCI, indicating that other biological pathways may be involved in addition to those well-known (Table 2A and Figure 2).

## Discussion

Currently, MCI has become a broad construct defined as a clinical syndrome with multiple clinical profiles due to various etiologies (Hulette et al., 1998; Bruscoli and Lovestone, 2004). MCI is considered an intermediate stage of cognitive impairment that may be a transitional phase before dementia (Petersen et al., 2014). Up to this day, the diagnosis of MCI is based on clinical criteria and evaluation and therefore relies on the evaluator's expertise, the neuropsychological tools applied, and the characteristic of the patient (Dunne et al., 2021).

Some neuropsychological profiles could provide some information about the underlying etiology of the MCI (Petersen et al., 1999) and the risk of progression to dementia (Petersen et al., 2001) or regression to normal cognition (Koepsell and Monsell, 2012). However, the information provided solely by the neuropsychological assessment is not enough to give an adequate risk assessment, and therefore some authors suggest complementing this information with the use of biomarkers (Campbell et al., 2013; Dunne et al., 2021). In this context, peripheral biomarkers could add or provide information on the possible etiology of the MCI, the individual risk of progression and help to decide about potential treatments.

Within the study of biomarkers, it is crucial and necessary to work with (i) an easily accessible sample and (ii) a stable, quickly obtained, sensitive, and reproducible biomarker (Lashley et al., 2018). miRNAs obtained in blood meet perfectly with these conditions and are therefore of great interest for implementation as diagnostic or prognostic biomarkers (Swarbrick et al., 2019).

Additionally, miRNAs can regulate several signaling pathways *via* multiple and diverse targets in response to pathophysiological stimuli making them also potentially attractive candidates with therapeutic interest. As we described, numerous dysregulated miRNAs have been detected in biofluids from MCI patients. Some of these miRNAs participate in the regulation of Aβ generation, synaptic dysfunction, and Tau phosphorylation (Lu et al., 2019). miRNA profiles are known to be altered in several regions of the brain in AD; however, which miRNAs are associated with early-stage AD remains uncertain. Here, we performed a systematic review of miRNAs reported to date in peripheral fluids in MCI cases. In addition, we specifically focused our analysis on the association between each reported miRNA and modulation of AD-associated target genes since its deregulation may reflect critical pathological changes in the brain.

Prior studies have noted that members of a miRNA family exhibit similar functions due to conserved motifs, such as seed sequences (Gardner et al., 2009; Kamanu et al., 2013). This insight provides an interest in comprehensively understanding the function of family-specific mature miRNA, their co-expression, and finding possible inter-miRNA family relationships between miRNAs and specific-target gene modulation (Kamanu et al., 2013). In line with our observation, of the 86 miRNAs levels reported in the 30 selected articles, 4 miRNAs families (mir-29, mir-30, mir-181, and mir-200) were found. Geekiyanage et al. showed significant correlations between MCI to AD progression status and mir-29a, mir-29b, and mir-181c in the regulation of *SPTLC1/2* gene expression, an enzyme involved in the modulation of ceramide levels, which is increased in the cerebral cortices of patients with sporadic AD (Geekiyanage et al., 2012). Furthermore, the mature sequence miR-29b-3p, product of mir-29b isomiR, was downregulated in plasma samples from aMCI subjects in a small Chinese cohort. Molecular evidence indicated that miR-29b-3p might be closely associated with AD pathology by regulating *BACE1* activity (He et al., 2021).

On the other hand, the mir-181 family has been extensively evaluated by several researchers. Agostini et al. have observed a significant correlation of miR-181a-5p levels and *SNAP-25* gene expression, and activity detected in blood in an Italian AD and MCI cohort (Agostini et al., 2019). The *SNAP-25* gene plays a pivotal role in the functional restoration of brain decline observed in neurodegenerative disease (Agostini et al., 2019). Similar results were found by Cai et al., who suggest that the interaction between miRNA and SNAP-25 expression could contribute to the alterations in synaptic functionality, activity, and neuroplasticity observed in the progression of MCI to AD (Cai et al., 2009). Siedlecki-Wullich et al. have shown an increase in miR-181c-5p plasma levels in both MCI and AD subjects. miR-181c-5p have two synaptic proteins as potential targets: neuronal pentraxin 1 (*NPTX1*) and neuronal pentraxin receptor (*NPTXR*) (Siedlecki-Wullich et al., 2019). These proteins are involved in synaptic homeostatic plasticity. Several authors have reported a transient increase in *NPTX1* and *NPTXR* levels in CSF samples in autosomal dominant AD, MCI, and early-stage AD cases (Ringman et al., 2012; Wildsmith et al., 2014; Llano et al., 2017; Duits et al., 2018). A fourth mir-181 family member was found to be upregulated in MCI before conversion to AD. mir-181 levels correlate with an increased CSF Aβ concentration and hippocampal atrophy, suggesting that mir-181c could be involved in the conversion from MCI to AD by regulating Fidgetin, B-cell lymphoma 2, and *SIRT1* genes expression (Ansari et al., 2019). In our integrated analysis of miRNA datasets, it must be noted that mir-181 family members did not show a direct correlation to AD-associated target genes. However, as several studies refer, its association to other targets involved in AD-associated pathways indicate that other biological process may be modulated in addition to those well-known (Cai et al., 2004, 2009; Agostini et al., 2019; Ansari et al., 2019; Siedlecki-Wullich et al., 2019).

Among all miRNA precursor families associated and reported in MCI, we observed one miRNA family upregulated, the mir-200 family. Guedes et al. have shown that mir-200b isomiR was upregulated in blood-derived monocytes and monocyte-derived macrophages samples from a large Portuguese population MCI (Guedes et al., 2016). mir-200b targets the 3' untranslated region of the *APP, Aβ*_42_, and *MGAT3* genes. Lu et al. reported that serum mir-200b levels in MCI subjects were significantly higher than in AD cases; however, the mir-200b level in AD subjects was lower concerning controls (Liu et al., 2021). This finding suggests that miRNA families encode more complex intrinsic features than conserved motifs, inter-miRNA family relationships, or imply shared roles in specific biological pathways and regulatory mechanisms that are not yet fully understood.

Regarding longitudinal studies, we observed a significant disadvantage related to the number of subjects analyzed in each study, with the number of subjects studied being <50 subjects with MCI (Sheinerman et al., 2013; Ansari et al., 2019; Kenny et al., 2019). Only one study stands out for analyzing a larger cohort of about 450 subjects with MCI (Xie et al., 2017). Longitudinal studies allow ROC curve analyses, which provide study accuracy, sensitivity, and specificity values that allow predicting the usefulness of these miRNAs as biomarkers capable of predicting the risk of progression from an altered cognitive state to dementia such as AD (Colijn and Grossberg, 2015). In this line, Xie et al. provided a prognostic role of circulating mir-206 in converting from aMCI to AD. According to the mir-206 predictors had as target genes *BDNF* and *SIRT1* genes, which showed that the expression levels of the genes were reduced while the levels of mir-206 were upregulated (Xie et al., 2017).

Genetic regulation is complex, including transcriptional, translational, and posttranslational processes, which are affected by miRNAs directly or indirectly (Kaczkowski et al., 2009; Kamanu et al., 2013; Mushtaq et al., 2016). miRNA targets can be validated experimentally *via* multiple molecular biology techniques. Here, we found that the most used techniques in the literature include qRT-PCR, sequencing, western blotting, miRNA microarrays, and *in situ* hybridization. In addition, we also found that most of the target genes regulated by the 86 miRNAs were *BACE1* and *APP*, two genes highly related to AD. Our dataset identified eight target genes in the same block that were functionally related to AD and dementia. Therefore, the miRNAs (mir-29a, mir-29b, mir107, mir-146a, mir-34c, and miR-130a-3p) in module highly correlated with AD could be considered interesting biomarkers for clinical screening and treatment of early-stage AD (Figure 3). This suggests that these miRNAs could play an essential role in regulating cognitive decline and progression to AD.

In order to further explore the validated target genes in the expression module of miRNAs, we selected four predicted target databases to perform the analysis for the miRNAs with validated target genes related to AD or dementia. Empirical validation of miRNAs target is time-consuming and expensive; hence, computational approaches to predict targets have also been developed, such as Tarbase (Papadopoulos et al., 2009), myRTarBase (Hsu et al., 2011), myRecords (Xiao et al., 2009), and Targetscan (Csardi, 2013). The validation methods used to demonstrate the physical interaction between the miRNAs and the target include luciferase reporter assay, PAR-CLIP, CLASH, and HITS-CLIP (Nicolas, 2011). Due to the large volume of data, we selected only those targets validated by the standard gold method (luciferase reporter assay). Among the blue modules, the miRNAs of the target genes involved in AD were mir-107, mir-29b, and miR-130a-3p, whereas red modules, the mir-135, miR-659-3p, miR-491-5p, miR-483-5p, and mir-206, are associated with targets related to other dementias (Figure 4). These results suggest that there may be miRNAs related to the early stages of AD. According to our analysis, the mir-181 family, miR-29a-3p, miR-146a-5p, miR-34c-5p, and miR-130a-3p, could be potential biomarkers; however, more research needs to be done to verify these miRNAs as possible biomarkers as well as the target genes they regulate and the neurobiological pathways involved.

## Concluding Remarks

A promising biomarker is the one that is technically easy to measure, is obtained *via* non-invasive techniques, has an excellent sensibility to represent the progression status of a disease or treatment-associated changes as well as specificity to inform a differential diagnostic (Gupta et al., 2013; el Kadmiri et al., 2018). Biomarkers could also confirm a diagnosis or classify a specific cluster of patients, for example, MCI subjects (Lista et al., 2013; Kayano et al., 2016). In this scenario, miRNAs have become promising alternatives because (i) miRNAs can be detected from plasma, urine, saliva, CSF, extracellular fluid, and tissues, (ii) cell-free miRNA are stable in blood samples (Tribolet et al., 2020), (iii) evidence indicates that miRNA expression patterns reflect some aberrant conditions, and sometimes, it shows a time-progression response (Keller et al., 2014), and (iv) miRNAs can be easily detected with qRT-PCR with no considerable time-consuming protocols and economic considerations.

Despite the advances in the biological role of miRNAs, some technical difficulties complicate the transfer of these small molecules to clinical setups as biomarkers. Some problems arise from the high conservative nature of some miRNA families (Kaczkowski et al., 2009), the low concentration of these molecules in biological fluids (Kayano et al., 2016; Yang et al., 2018; Liu et al., 2021), and that generally there is no single miRNA associated with a tissue-specific condition (Mushtaq et al., 2016; Swarbrick et al., 2019); it is more a pattern expression condition, hence it no feasible to use a single miRNAs as a biomarker (He et al., 2021).

Another important factor is that the plasma concentration of brain-enriched miRNA can originate from different sources. Of the total miRNA known to date, ~70% are expressed in the brain, spinal cord, or peripheral nerves (Adlakha and Saini, 2014) where they play key roles in processes such as neurogenesis (Stappert et al., 2018), synaptic transmission (McGowan et al., 2018) synaptic plasticity (Ye et al., 2016), regulation of the blood-brain barrier integrity, and disease (Ma et al., 2020). Some miRNAs are highly expressed in specific neuronal compartments, such as axons, dendrites, and synapses (Kye et al., 2007), where they are essential for normal neuronal function and survival (Schratt, 2009). Furthermore, dysregulated miRNA levels have been previously linked to impaired learning, memory, and cognition (Wang et al., 2012). Despite that miRNAs are differentially expressed in certain cell types, organs and tissues in many human pathologies (Hua et al., 2009) it is well-known that miRNAs expressed in the brain can cross the blood-brain barrier and be released into the blood circulatory system. In this context it is reasonable to think that changes in the expression of these miRNA in the blood? can be used to monitor the neurodegeneration that occurs in the brain.

Additionally, the normalization of circulating miRNA concentration is fundamental to infer the technical variability between samples (i.e., the difference in sample volume, processing time, PCR yield, library composition). The normalization strategy depends on which experimental methodology is used to evaluate miRNAs. For qPCR, which is highly used as a platform for the validation of biomarkers, normalization relies on exogenous and endogenous controls or against the total levels of miRNA (Huggett et al., 2005). Nevertheless, the Minimum Information for Publication of Quantitative Real-Time PCR Experiments (MIQE) guidelines indicate that the preferred way to normalize is by using internal reference genes that have the same nature as the molecule being analyzed, hence it is preferable to use two or more endogenous miRNAs as reference genes rather than exogenous miRNAs or lncRNAs. It is important to note that no one-fits-all miRNA endogenous control can be used for every tissue or sample analyzed, so researchers must validate its reference controls in each setting. There are multiple software available that calculate the stability score of each reference controls that can be used to evaluate which control is more stable and which combination of miRNAs have less inter- and intragroup variability (for more information, see BestKeeper, NormFinder, and GeNorm algorithms). Once reference controls are selected, the geometric average of the quantification cycle (Cq) of the endogenous controls is used to normalize the samples. Afterward, the relative expression of miRNAs can be calculated using the ΔΔCq methodology. For high-throughput methodologies, used principally in the screening phase of biomarker discovery, the normalization is based on mathematical approaches, whereas microarrays can be normalized using the mean expression of all miRNAs detected or a quantile normalization.

In the same line, other controls necessary when using plasma and serum samples are hemolysis controls, as hemolysis has been described to alter the detection of circulating miRNAs. Hemolysis can be assessed by either spectrophotometer reads or ratios of specific miRNAs (Shah et al., 2016). Regarding the compensation of biological differences (e.g., changes in microRNAs explained by the permeability of the blood-brain barrier), these are not compensated and in fact, these differences are used to detect a pattern of expression that is putatively the base of a miRNA diagnosis panel. However, in order to make a correct differential diagnosis we need to take into account that MCI can be a precursor to AD, but not all MCI patients develop AD. Furthermore, some MCI patients remain stable, convert back to normal, or progress to non-AD disease, therefore biomarkers with good prediction capacity have been developed using longitudinal studies to evaluate MCI that convert to AD, from those who do not (Shigemizu et al., 2020).

miRNAs are involved in neural development and differentiation; thus, it appears to have a potential role in the development of neurodegenerative diseases such as AD (Moradifard et al., 2018). Since one miRNA could regulate many genes as targets, and those targets can be regulated by several miRNAs, studying the miRNA profile in multifactorial diseases such as AD appears to be a potential advantage (Iqbal and Grundke-Iqbal, 2010). We are confident that the study of the miRNA expression profile could avoid the condition where genes can be up or downregulated by miRNAs. In addition, relationships have been found between the target genes of the dysregulated miRNAs in patients with AD and the biological pathways related to this disease (Moradifard et al., 2018).

On the other hand, in this review we have shown that there are dysregulated miRNAs in early stages of AD such as MCI. Due to the reported relationship between these miRNAs and genes related to AD, specifically *BACE1, MAPT, SNAP-25, SPTLC1/2, TGF -*β*1*, and *APP* (Geekiyanage et al., 2012; Kumar et al., 2017; Nagaraj et al., 2017; Agostini et al., 2019), establishing a link between these genes and the differential levels of miRNAs at early stages of AD, would allow us to understand their association with biological pathways related to the disease. This link will provide us with an important insight that could considerably contribute to the early diagnosis of AD and the development of preventive therapies.

In summary, we identified five miRNAs expression profiles from peripheral fluid in MCI cases reported in 30 articles, with target genes related to AD. The relationship between AD modules and miRNAs related to MCI show possible candidate miRNAs that may be exploited as fluid biomarkers or therapeutic targets of early-stage AD. However, additional research needs to be used to verify these miRNAs, the target genes they regulate, and the neurobiological pathways involved.

## Data Availability Statement

The original contributions presented in the study are included in the article/supplementary material, further inquiries can be directed to the corresponding author/s.

## Author Contributions

NO, PO, and CD-A developed the study concept and the study design. NO, SS, and PO performed PRISMA analysis. NO and PO performed data analysis and interpretation under the supervision of CD-A. NO, SS, CA, TL, PO, NC-V, CV, and CD-A drafted the manuscript and discussed contributions from all co-authors. MR, CA, and AB provided critical revisions. All authors have participated sufficiently in work and approved the final version of the manuscript for submission.

## Funding

This work was funded by the Alzheimer's Disease Association 2018-AARG-591107, ANID/FONDEF ID20I10152, ANID/FONDECYT 1210622, and Anillo ACT210096 to CD-A. PO was fully supported by the fellowship of Social Neuroscience and Cognition Ph.D. program from Universidad Adolfo Ibanez.

## Conflict of Interest

NC-V, CV, and AB were employed by company Neurognos Spa. The remaining authors declare that the research was conducted in the absence of any commercial or financial relationships that could be construed as a potential conflict of interest. The reviewer EJ-M declared a shared affiliation with one of the authors, TL, to the handling editor at time of review.

## Publisher's Note

All claims expressed in this article are solely those of the authors and do not necessarily represent those of their affiliated organizations, or those of the publisher, the editors and the reviewers. Any product that may be evaluated in this article, or claim that may be made by its manufacturer, is not guaranteed or endorsed by the publisher.
